# Self-Powered Well-Aligned P(VDF-TrFE) Piezoelectric Nanofiber Nanogenerator for Modulating an Exact Electrical Stimulation and Enhancing the Proliferation of Preosteoblasts

**DOI:** 10.3390/nano9030349

**Published:** 2019-03-03

**Authors:** Aochen Wang, Ming Hu, Liwei Zhou, Xiaoyong Qiang

**Affiliations:** School of Microelectronics, Tianjin University, Tianjin 300072, China; 13602176911@163.com (L.Z.); shawn_q@tju.edu.cn (X.Q.)

**Keywords:** well-aligned nanofibers, P(VDF-TrFE), piezoelectric nanogenerator, preosteoblasts electrospinning

## Abstract

Electric potential plays an indispensable role in tissue engineering and wound healing. Piezoelectric nanogenerators based on direct piezoelectric effects can be self-powered energy sources for electrical stimulation and have attracted extensive attention. However, the accuracy of piezoelectric stimuli on piezoelectric polymers membranes in vitro during the dynamic condition is rarely studied. Here, a self-powered tunable electrical stimulation system for assisting the proliferation of preosteoblasts was achieved by well-aligned P(VDF-TrFE) piezoelectric nanofiber membrane (NFM) both as a nanogenerator (NG) and as a scaffold. The effects of electrospinning and different post-treatments (annealing and poling) on the surface wettability, piezoelectric β phase, ferroelectric properties, and sensing performance of NFMs were evaluated here. The polarized P(VDF-TrFE) NFM offered an enhanced piezoelectric value (d_31_ of 22.88 pC/N) versus pristine P(VDF-TrFE) NFM (d_31_ of 0.03 pC/N) and exhibited good sensing performance. The maximum voltage and current output of the P(VDF-TrFE) piezoelectric nanofiber NGs reached −1.7 V and 41.5 nA, respectively. An accurate electrical response was obtained in real time under dynamic mechanical stimulation by immobilizing the NGs on the flexible bottom of the culture plate, thereby restoring the real scene of providing electrical stimulation to the cells in vitro. In addition, we simulated the interaction between the piezoelectric nanofiber NG and cells through an equivalent circuit model. To verify the feasibility of P(VDF-TrFE) nanofiber NGs as an exact electrical stimulation, the effects of different outputs of P(VDF-TrFE) nanofiber NGs on cell proliferation in vitro were compared. The study realized a significant enhancement of preosteoblasts proliferation. This work demonstrated the customizability of P(VDF-TrFE) piezoelectric nanofiber NG for self-powered electrical stimulation system application and suggested its significant potential application for tissue repair and regeneration.

## 1. Introduction

Electrical stimulation is widely used to compensate for the altered electrical communication in diseased tissue and thus improve tissue regeneration [1,2,3]. External electric fields can improve physiological strength and can guide cell-orientated growth and influence cell proliferation and differentiation including for nerves, cardiac cells, and osteoblasts [4,5,6,7,8,9,10]. However, traditional electrical stimulator requires invasive microelectrodes, an external power supply, and electrical wires. This is very uncomfortable, inconvenient, and unreliable. Thus noninvasive, wireless, portable, self-powered, and wearable electronic devices are urgently needed for electrical stimulation system. Recent work in nanogenerators (NGs) has exhibited significant progress in noninvasive and self-powered electrical stimulation [8,11]. In the framework, piezoelectric nanogenerators (PENGs) based on piezoelectric polymers that can generate electric surface charges under external mechanical vibration and thus achieve cordless electrical stimulation, have attracted a lot of attentions [12,13,14,15]. The electrical output produced by the PENG acts as an electrical stimulation signal whose value is related to the piezoelectric property of the material [16,17]. One such material is poly(vinylidene fluoride-trifluoroethylene) (P(VDF-TrFE)) with outstanding piezoelectric properties due to steric hindrance from the extra fluorine atoms in the TrFE inducing an all-trans stereochemical configuration [18,19]. It is well-known that the β phase is the most highly polar crystalline phase of PVDF and its copolymers [20]. Accordingly, P(VDF-TrFE) with a high content of piezoelectric β-crystalline offers excellent piezoelectric properties. 

The electrospinning process can produce piezoelectric fibers by stretching as well as in situ poling during the fabrication process, paving the way for piezoelectric nanofiber NGs [21,22,23,24]. These electrospun aligned fibers have a higher content of β-crystalline phase versus random fibers [25]. In addition, various post-treatments, such as annealing and poling treatments, can produce piezoelectricity via dipole orientations; however, the influence of poling treatments on the β-crystalline phase content is rarely studied [26]. Therefore, to further improve the piezoelectricity of electrospun fibers and thus enhance the performance of PENG, it is critical to understand the specific significance of post-treatments on the piezoelectric performance [27,28].

In addition to its good piezoelectric properties, P(VDF-TrFE) also offers easy processing and nanomaterial formability. Therein, electrospinning has been widely employed to fabricate the micro-/nanofibers in ordered, random, and specific patterns. The electrospun fibrous films have a high surface-area-to-volume ratio similar to the structure and characteristics of extracellular matrix (ECM) [29]. In this framework, research has demonstrated that the alignment of nanofibers can structurally mimic the parallel orientation of the tissues and modulate cell adhesion, migration, proliferation, and differentiation [30,31,32,33]. 

Kai et al. prepared electrospun aligned and randomly oriented poly(e-caprolactone)/gelatin (PG) scaffolds. They found that the aligned PG scaffold could enhance the cells attachment and alignment [34]. Hitscherich et al. reported that mouse embryonic stem cell-derived cardiomyocytes (mES-CM), cultured on the aligned P(VDF-TrFE), were aligned along the fibers and expressed classic cardiac-specific markers [35]. Therefore, based on its piezoelectric property and processability, P(VDF-TrFE) has been widely used for biomedical scaffolds for tissue engineering and electrical stimulation. Genchi et al. fabricated P(VDF-TrFE)/BaTiO_3_ composite films as a substrate for piezoelectric stimulation to enhance the differentiation of neuroblastoma cells [36]. Later Deng’s group used the BaTiO_3_/P(VDF-TrFE) nanocomposite membrane and leveraged the piezoelectric properties to promote bone regeneration [37]. However, the exact value of electrical stimulation induced by the piezoelectric substrate is not clear when external mechanical vibration is also applied to the cells in vitro. Thus, to better understand the effect of piezoelectric regulation on cell behavior, there is a need to measure this electrical stimulation.

In this work, we explored the effects of the exact electrical signals generated by the P(VDF-TrFE) piezoelectric nanofibers NGs on the proliferation fate of preosteoblasts. Here, the fabrication of self-powered piezoelectric nanofiber NG used as cell scaffold was based on electrospun well-aligned P(VDF-TrFE) nanofiber membranes (NFMs). The effects of annealing and poling post-treatments on the surface wettability, piezoelectric β phase, piezoelectricity, and sensing performance of P(VDF-TrFE) NFMs were investigated. In order to study the dependence of the electrical outputs of NGs on the degree of polarization, two kinds of NGs processed by different poling electrical fields were prepared. In particular, they were fixed to the flexible bottom of the culture plate, and the accurate electrical response was measured in real time under dynamic mechanical stimulation, thereby restoring the real scene of the electrical stimulation of the cells in vitro. In addition, we simulated the interaction between piezoelectric nanofiber NG and cells through an equivalent circuit model. In order to study the role of NFMs as a scaffold, the effects of well-aligned and random interfaces of NFMs on the morphology of preosteoblasts were investigated. The well-aligned nanofibrous platforms could guide and elongate the cells. Finally, we compared the effects of different outputs stimulation of P(VDF-TrFE) nanofiber NGs on cell proliferation in vitro by applying a dynamic piezoelectric stimulus. This work demonstrates a significant potential of P(VDF-TrFE) piezoelectric nanofiber NG as self-powered electrical stimulation system for assisting tissue repair and regeneration.

## 2. Materials and Methods

### 2.1. Electrospinning of Nanofiber Membranes (NFMs)

The P(VDF-TrFE) (75/25 mol%, Piezotech Inc., Pierre-Bénite, France) nanofibers were prepared as described previously [38]. Briefly, P(VDF-TrFE) powders were dissolved in the N,N-dimethylformamide (DMF) and acetone mixture solution (6:4 v/v) at 20% (w/v). The P(VDF-TrFE) spinning solution was injected into a 5-ml syringe fitted with a 22 G needle. A syringe pump (KDS101, KD Scientific, Holliston, MA, USA) was used to supply a constant flow rate of 1 mL/h. A high voltage of 15 kV was applied between the tip of the syringe needle and the grounded roller collector at a distance of 10 cm. The thickness of the electrospun NFM was regulated at ~30 µm by controlling the electrospinning time. The electrospun NFMs were dried at 65 °C for 10 h to volatilize the residual solvent. The annealed NFMs were kept in a vacuum oven at 135 °C for 4 h. The poled samples were pressed via a powder compression machine (BJ-15, BOJUNKEJI Inc., Tianjin, China) after annealing, in order to ensure no conduction between subsequent sputtering electrodes. Next, the samples were prepared by sputtering gold electrodes on both the top and bottom surfaces, then placed in a fixture and immersed completely in silicon oil. Afterwards, the silicon bath was heated to 115 °C by a hot plate (HCT basic, IKA Inc., Staufen, Germany) and then a polarization electric field of 80 to 100 MV/m was applied. After 30 min of thermal poling treatment, the polarization voltage was kept constant, cooled down to room temperature and finally the voltage was removed. Next, pristine P(VDF-TrFE) NFMs without any postprocessing (annealing and poling) were labeled as U-NFM, and the samples treated by annealing were coded as A-NFM. The poled samples were denoted as P-NFM. The NFMs poled with the electric field of 80 MV/m and 100 MV/m were labeled as P80-NG and P100-NG, respectively.

### 2.2. Characterization and Measurements of NFMs

The morphology of electrospun NFMs was observed with a scanning electron microscope (SEM, SU8020, Hitachi Ltd., Tokyo, Japan) at an accelerating voltage of 5 kV. ImageJ software (National Institutes of Health, Bethesda, USA) was used to analyze the mean fiber diameter. Tensile testing was done with a tensile test machine (ESM301, Mark-10, Copiague, NY, USA) at room temperature with a cross-head velocity of 10 mm/min. The sample was cut to dumbbell shape (10 mm long and 5 mm wide). The contact angles were recorded employing contact angle goniometer (XG-CAMB1, Xuanzhun co., Ltd, Shanghai, China) by sessile drop method at room temperature. A droplet of deionized water was dropped from the capillary mouth to stop on the membrane surface and the angle of the droplet on the upper surface of the membrane was collected and analyzed. X-ray diffraction (XRD) patterns were done on an X-ray diffractometer (X′pert^3^ Powder, PANalytical Ltd., Almelo, The Netherlands) and recorded over an angular range from 10° to 50°. Infrared spectra were recorded on a Fourier transform infrared spectrometer (FTIR, VERTEX80v, Bruker Corp., Billerica, MA, USA) from 400 cm^−1^ to 1600 cm^−1^. The polarization-electric field (P-E) hysteresis loops were obtained by precision multiferroic and ferroelectric test systems (Radiant Technologies Inc., Alpharetta, GA, USA ) under a unipolar electric field at a measurement frequency of 10 Hz.

The dynamic piezoelectric coefficient d_31_ was determined with a homemade measurement system nearly identical to setup described previously [39]. The output voltage of the samples during the process of stretching–relaxing was recorded with a DSP lock-in amplifier (SR830, Stanford Research Systems, Sunnyvale, CA, USA). The piezoelectricity coefficient d_33_ was measured using a quasi-static d_33_ measuring instrument (Institute of Acoustics, Chinese Academy of Science, ZJ-4AN, Beijing, China).

### 2.3. Measurements and Stimulation of Piezoelectric Nanofiber Nanogenerators (NGs)

The generated voltage, current, and charge of the NGs generated via dynamic mechanical stimulus from a speaker (8 Ω, 1 W) were assessed by a Tektronix Keithley electrometer 6514. The speakers were driven by sinusoidal signals including a 4 V amplitude at different frequencies (2 Hz, 3 Hz, 4 Hz, and 5 Hz) generated by function generator (DS345, Stanford Research Systems, Sunnyvale, CA, USA).

To quantitatively analyze the deformation and potential distribution of P(VDF-TrFE) nanofibers based on NG by a uniaxial stress (~0.5 Mpa), we performed finite element modeling (FEM) by COMSOL multiphysics software (5.3, COMSOL Inc., Stockholm, Sweden).

From the analysis of electrical characteristics, the cell membrane can be equivalently modeled as a resistor capacitor with electrical properties such as extracellular medium resistance (R_cm_), membrane resistance (R_m_), membrane capacitance (C_m_) and ion equilibrium potential (V_m_). According to these four characteristics, an equivalent circuit can be constructed [40,41]. The external electrical stimulation is generated by a P (VDF-TrFE) piezoelectric nanofiber NG under a dynamic mechanical vibration, which can be represented as a voltage source. By using the equivalent circuit model, the expected behavior of the effective voltage and current applied to the cell membrane by NG was evaluated. The circuit was simulated by Multism software (14.0, National Instruments Co., Austin, TX, USA).

### 2.4. Cell Culture

MC3T3-E1 cells (Subclone 14, mouse preosteoblasts, Innochem Ltd., Beijing, China) were cultured in Alpha Minimum Essential Medium (α-MEM, Gibco) with 2 Mm L-glutamine (Gibco) and 1 mM sodium pyruvate (Gibco) supplemented with 10% fetal bovine serum (Gibco), 100 U/mL penicillin, and streptomycin (Gibco) in a humidified atmosphere with 5% CO_2_ in air at 37 °C.

### 2.5. Cell Alignment Quantification

The cell alignment on the NFM was quantified by a two-dimensional fast Fourier transform (2-D FFT) image analysis method [42]. Briefly, the fluorescent cell photographs were converted to 8-bit grayscale and then cropped a 1024 × 1024 pixel square. This was then masked with a transparent circular pattern, and the corners were filled with black. The processed photograph was analyzed by FFT function in ImageJ software [43]. Pixel intensities along the radian were summed using the oval profile plug-in and normalized by the lowest intensity value [44]. The normalized results represent the percentage of cells aligned along a certain direction.

### 2.6. Piezoelectric Stimulation and Cell Proliferation Assay

The dynamic piezoelectric stimulus used the custom-made speakers that provided uniform mechanical vibration to cells in monolayer cultures. Specialized flexible-bottomed culture plates (BF-3001U, Flexcell Int. Co., Austin, TX, USA) made of silicone elastomer membrane were used to culture the cells. The strain applied to the silicone elastomer membrane was directly transmitted to the NGs to generate piezoelectricity. The synthesized function generator and power amplifier were used to control the frequency and amplitude of deformation applied to the culture plate (experimental vibration frequency: 2 Hz; amplified voltage: 4 V).

To clarify the feasibility of P(VDF-TrFE) nanofiber NGs as exact electrical stimulation and demonstrate the effects on the proliferation of MC3T3-E1 cells, P100-NG and P80-NG were selected as the experimental groups, and the nonpiezoelectric A-NFM served as the control group. The cells were seeded at a density of 2 × 10^4^ cells per well on the various NGs. The piezoelectric stimulation was applied to MC3T3-E1 cells for 30 min per day for 1 day, 3 days, or 5 days. The proliferation of the cultured MC3T3-E1 cells was measured using the cell count kit-8 (CCK-8, Dojindo Molecular Technology). The culture medium was first replaced with 1.5 mL α-MEM medium plus 10% CCK-8 solution. After 4 h incubation at 37 °C, the production of water-soluble formazan dye was determined using a microplate reader (MULTISKA NMK3, Thermo Fisher Scientific, Waltham, MA, USA) at a wavelength of 450 nm. The culture medium was changed every 2 days. Three parallel replicates were examined each time for each group.

To observe the cell morphology on the NFMs, the MC3T3-E1 cells were fixed with 4% paraformaldehyde solution in PBS (Sigma) for 10 min and then washed three times with warm 1× PBS and blocked with 1% bovine serum albumin (BSA, Sigma) solution for 60 min. The cytoskeleton was stained with Phalloidin (Invitrogen) conjugated to Alexa Fluor 488 (1:200 diluted) for 2 h at 37 °C, and the nucleus was stained with 4’,6-diamidino-2-phenylindole (DAPI, 300 nM, Life Technology) for 10 min.

### 2.7. Statistical Analysis

The data were expressed as the mean ±standard deviation (SD). Statistical analysis was determined using one-way ANOVA. Statistical differences were tested with a one-way ANOVA using the *t*-test (Tukey test) for independent samples. Statistical significance was accepted at * *p* < 0.05 and ** *p* < 0.01.

## 3. Results and Discussion

### 3.1. Morphology and Characterization of NFMs

**Figure 1:**
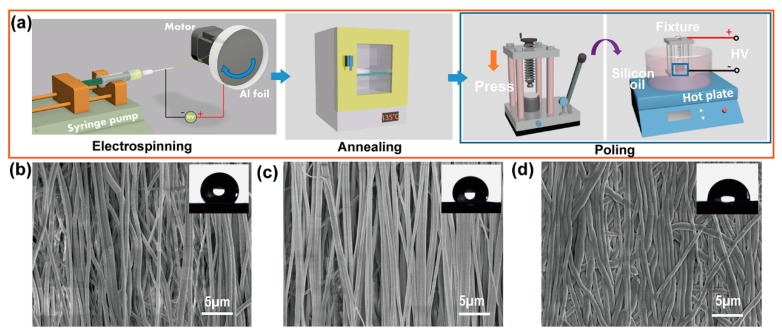
The morphology and contact angle of electrospun nanofiber membranes (NFMs). (**a**) Schematic illustration of the preparation and treatment of different samples. Scanning electron microscope (SEM) micrographs of (**b**) U-NFM, (**c**) A-NFM, and (**d**) P-NFM. The insets represent the contact angles corresponding to U-NFM, A-NFM, and P-NFM, respectively. (U-NFM represented pristine poly(vinylidene fluoride-trifluoroethylene)(P(VDF-TrFE)) NFM without any postprocessing; A-NFM represented annealed P(VDF-TrFE) NFM; P-NFM represented the poled samples.)

Figure 1 shows a schematic for the electrospinning process and post-treatment process. The P(VDF-TrFE) nanofibers were fabricated with the optimized electrospun parameters using the electrospinning setup. They had a strongly aligned and uniform morphology (Figure 1b). The mean diameter of electrospun nanofibers was 590 nm ± 26 nm with further size analysis in Figure S1a. After annealing (Figure 1c and Figure S1b), several microvoids appeared clearly on the surface of the nanofibers and the surface of single fiber became rough. Here, the A-NFM exhibited a porous structure and a higher surface-area-to-volume ratio than the U-NFM, which is beneficial for the migration of implanted cells. Although the P-NFM exhibited the flat surface owing to the mechanical pressing to make the nanofibers flattened, it still had nanofibrous surface. As shown in the insets of Figure 1b–d, the average contact angle of U-NFM was 129.13°. Although the single nanofiber surface of A-NFM was rougher than that of U-NFM, the contact angle of A-NFM decreased to 113.38°. This phenomenon can be explained as follows. In addition to the influence of surface roughness, the contact angle is also related to the surface free energy [45]. The annealing treatment resulted in the transformation of some nonpolar α phases into polar β phase. The presence of polar β phase increased the dipolar interaction between the NFM and water molecules, which increased surface energy and reduced the contact angle [46,47]. The influence of high surface energy on the contact angle of A-NFM is greater than that of surface roughness. The P-NFM had the smallest contact angle (91.14°) due to the combined effects of nanostructure flattening and polar β phase, demonstrating the surface wettability of P(VDF-TrFE) NFM can be improved by poling treatment. In addition to the highly-aligned and porous properties, the fibers of the polarized P-NFM samples were arranged more closely, and the fibers were bonded to each other. This improves the material’s mechanical properties (Figure 1d) [48]. Representative stress–strain plots are displayed in Figure S2. The mean elastic modulus of U-NFM, A-NFM, and P-NFM were 0.148 GPa, 0.426 GPa, and 0.876 GPa, respectively. This result proves that the poling treatment can significantly affect the mechanical robust. A well-aligned and uniform P-NFM with favorable mechanical properties shows its potential as a scaffold in tissue engineering.

**Figure 2:**
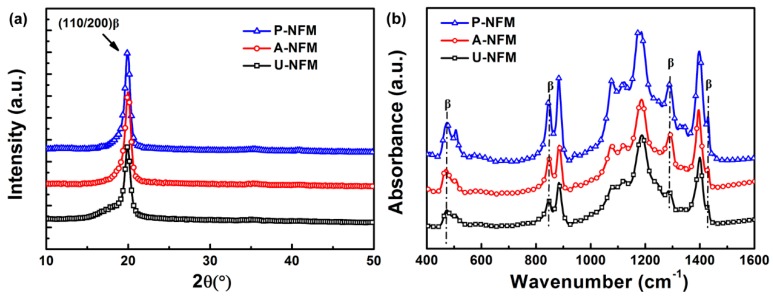
Crystalline characterization of P(VDF-TrFE) NFMs treated with different postprocessing steps. (**a**) X-ray diffraction (XRD) patterns and (**b**) Fourier transform infrared spectroscopy (FTIR) spectra.

The crystallinity of P(VDF-TrFE) NFMs treated with different postprocessing steps was determined via the XRD patterns (Figure 2a). There was a distinct reflection peak from 19 to 21 degrees for all NFMs corresponding to the diffraction of plane (200)/(110) of the β phase crystal [49,50]. The broad shoulder at ~18 degrees for U-NFM is associated with the amorphous phase. After annealing treatment, the shoulder completely disappeared for both A-NFM and P-NFM, but the diffraction intensity of the (200)/(110) plane increased. In addition, the increase in diffraction peak intensity of P-NFM versus A-NFM demonstrates that the β phase is enhanced after poling. By fitting the XRD patterns [51], the diffraction curve could be resolved into three regions: amorphous as well as α- and β-crystalline phases. Thus, α and β phases could be measured. The percentage of α phase in the U-NFM was 23.3% while that of the A-NFM was 26.5%. The P-NFM had less α phase—this might be due to the phase transformation from α-crystal phase to β-crystal phase after poling treatment. The percentage of β phase in the U-NFM and A-NFM was 43.1% and 46.6%, respectively; it was 69.2% in P-NFM.

The crystal phase structures could also be characterized by FTIR spectra (Figure 2b). The characteristic absorption bands [52,53] at 506, 840, 1285, and 1430 cm^−1^ are recognized as β phase structures whereas the absorbance peaks of the α phase structure appears at 532, 614, 765, 870, and 976 cm^−1^. Versus U-NFM, the intensity of characteristic bands corresponding to β phase increased for the A-NFM, which suggests that the annealing treatment improves the β phase content. Furthermore, the highest peak intensity of β phase was seen with P-NFM. This phenomenon is mainly due to thermal poling that increases with the degree of dipole orientation and phase transition of the β phase. There are fewer crystalline defects and enhanced β-crystallinity.

The XRD patterns and FTIR spectra indicate that an annealing treatment can slightly increase the β phase content while the thermal poling treatment can significantly improve the β phase crystallinity.

### 3.2. Effect of Postprocessing on NFM Piezoelectric Properties 

**Figure 3:**
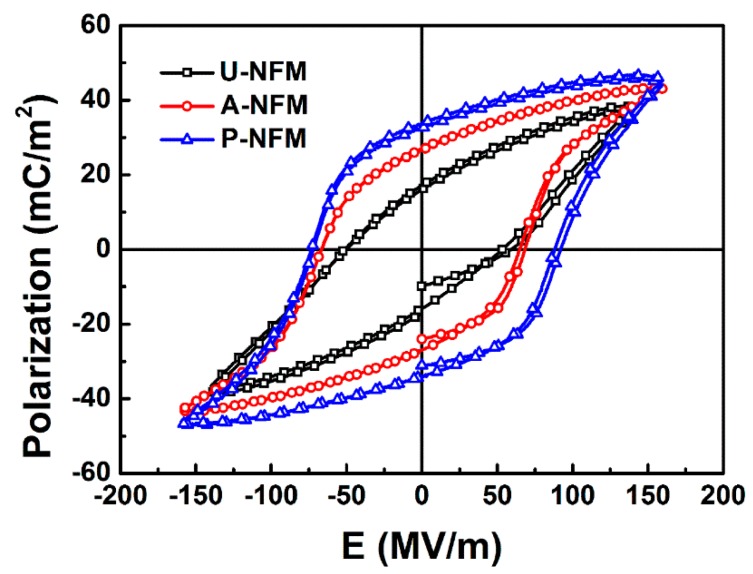
The P-E hysteresis loops of P(VDF-TrFE) NFMs treated with different postprocessing at a polarization electric field of 160 MV/m.

The polarization-electric field hysteresis loops (P-E loops) of U-NFM, A-NFM, and P-NFM at various electric fields are presented in Figure 3 and illustrate the ferroelectric behavior of nanofiber membranes treated with different postprocessing steps. The remnant polarization (Pr) and the saturated polarization (Ps) of U-NFM was 17.1 mC/m^2^ and 37.9 mC/m^2^, respectively (Figure 3a). After annealing, the Pr and Ps of A-NFM could reach 26.9 mC/m^2^ and 43.1 mC/m^2^, respectively (Table 1 and Figure 3b), indicating that the annealing process can increase the crystallinity. The Pr is mainly associated with a highly polar β-crystalline phase. Table 1 and Figure 3c show that a higher Pr of 32.6 mC/m^2^ could be obtained by P-NFM under a polarizing electric field of 160 MV/m. In addition, Pr was closer to Ps in sample P-NFM, and the P-E loops tended to be saturated. This suggested that the ferroelectric domain trends toward a single-domain. In particular, the higher Pr mainly originated from a β phase crystal domain reflecting the better ferroelectric properties. This result suggests that thermal poling can increase the β-crystalline phase content. In addition, the coercive electric field (E_c_) increased from 60.9 MV/m for U-NFM and 65.2 MV/m for A-NFM to 88.1 MV/m for P-NFM, suggesting that the ferroelectric domain of the β-crystal phase is not oriented easily. Consequently, the P-NFM had a strong ability to maintain polarization and possessed excellent piezoelectric property.

**Figure 4:**
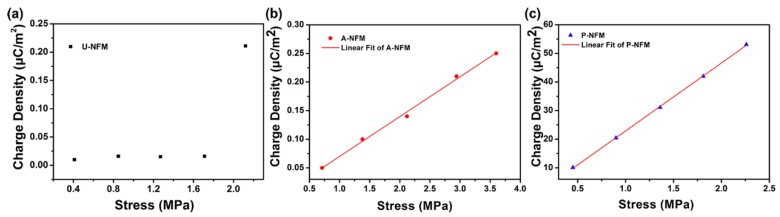
Experimental and theoretical studies of piezoelectric coefficient d31 of nanofiber membranes. (**a**) Experimental charge density–stress curve of U-NFM. Experimental (symbols) and linear fitting (lines) charge density–stress curves of (**b**) A-NFM and (**c**) P-NFM.

The relationship between charge density and applied stress is plotted in Figure 4 and can evaluate the piezoelectric coefficient d_31_. The slope of the line represents the piezoelectric coefficient d_31_ (Figure 4). The calculated d_31_ and the corresponding linear regression correlation coefficients (R^2^) are summarized in Table 1. The data illustrate that there is better agreement between the theoretical and experimental values of d_31_ for A-NFM and P-NFM than for U-NFM. The A-NFM and P-NFM showed good sensing performances. It means that P-NFM can provide accurate electrical stimulation under varying external stress and that P-NFM is more reliable in the application of NGs that provide electrical stimulation. 

In addition, there was almost no piezoelectricity in A-NFM (d_31_ = 0.07 pC/N) and U-NFM (d_31_ = 0.03 pC/N); however, P-NFM showed the highest piezoelectric coefficient (d_31_ = 22.88 pC/N, d_33_ = −31 pC/N) among all samples (Table 1). This is due to the fact that U-NFM has many amorphous crystalline phases, some nonpolar α phases and a small amount of polar β phases. With an annealing treatment, the amorphous phase of the nanofibers decreases, the total crystallinity increases and some nonpolar α phases are transformed to polar β phase, which increases the content of β phase and thus the remnant polarization was increased compared to U-NFM, nevertheless, the dipole orientation is disorderly, so the total spontaneous polarization is zero and the piezoelectric property is weak. After poling treatment, the transformation of the α phase to the β phase is further promoted leading to high remnant polarization and most electric dipoles are oriented along the direction of the externally applied electric field, underscoring the high piezoelectric performance. With the favorable mechanical and ferroelectric properties of P-NFM confirmed, we next used this material as piezoelectric nanofiber NG and evaluated the electrical response as well as the arrangement and proliferation of cells cultured on this material. 

### 3.3. Effect of Mechanical Stimulus on Electrical Performances 

**Figure 5:**
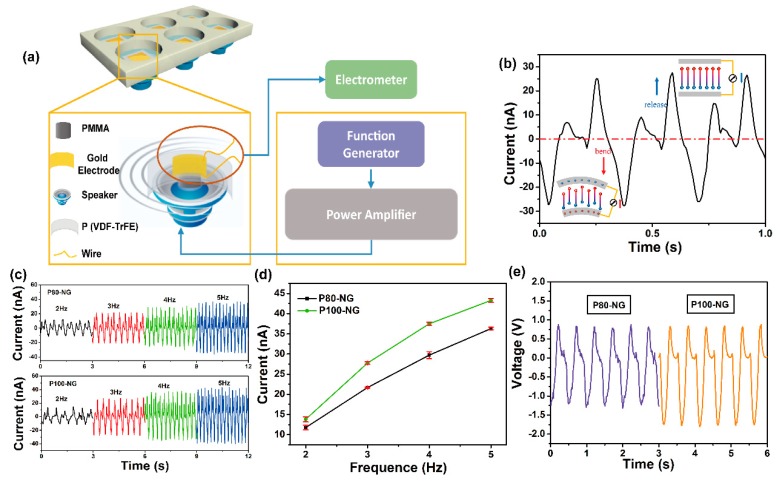
Effect of mechanical stimulus on electrical performances of different NGs. (**a**) Schematic diagram of the home-designed experimental shaker for providing periodic mechanical vibrations. (**b**) Piezoelectric output currents recorded during bending and releasing. The insets are the schematics of NG under mechanical bending deformation and releasing state, respectively, (P100-NG at a frequency of 3 Hz). (**c**) Current outputs of P80-NG (above) and P100-NG (below) in the frequency range of 2 to 5 Hz. (**d**) Comparison of measured current of P80-NG and P100-NG under different frequency from 2 to 5 Hz. (**e**) Voltage outputs of P80-NG and P100-NG at a frequency of 2 Hz.

To study the dependence of the electrical outputs of NGs on the degree of polarization, two kinds of NGs processed by different poling electrical fields were measured. Figure 5a shows the illustrative diagram of the experiment setup for imitating the real scene of the electrical stimulation of the cells in vitro during the dynamic mechanical vibration. The NG was deformed periodically via a self-designed setup driven with an amplitude of 4 V (peak-to-peak value). The resulting curve exhibited a periodic alternation of negative and positive responses corresponding to the deformed and released states of a piezoelectric nanofiber NG, respectively (Figure 5b). As the frequency increases from 2 Hz to 5 Hz, the generated peak-to-peak piezoelectric current increased from 18.1 nA to 39.7 nA in the P80-NG sample (Figure 5c upper); the P100-NG sample was modulated from 23.1 nA to 41.5 nA (Figure 5c lower). These results confirm that the charge transfer is kept equal at different vibration frequencies, but the output is due to rapid electron flow. 

Figure 5d shows that the measured current increased with increasing vibration frequency in both P80-NG and P100-NG. The slope of the P100-NG sample was 1.2-fold higher than that of P80-NG sample under the same vibration force. Figure 5e shows the results of induced voltage under a frequency of 2 Hz. The output voltage of P80-NG and P100-NG reached −1.3 V and −1.75 V, respectively. These results indicate that the electrical performance of the piezoelectric nanofiber NG are affected by poling treatment and the outputs can be modulated and optimized by adjusting the polarization treatment. In the following in vitro assay, piezoelectric nanofiber NG as an electrical stimulator can provide an exact stimulation to cells in real time during the dynamic status. 

### 3.4. Theoretical Modeling of NG-Cell Interaction

**Figure 6:**
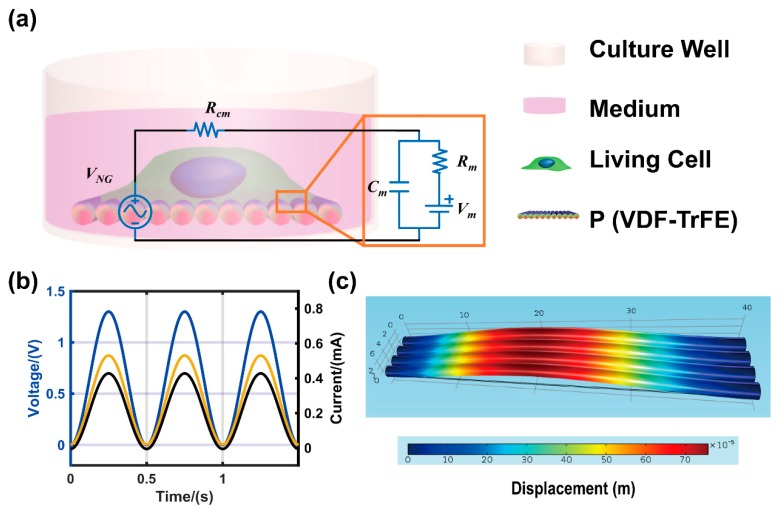
Electrical model of the NG-cell and FEM model of NG. (**a**) The diagrammatic sketch of NG-cell and corresponding equivalent circuit model. (**b**) The voltage (yellow) and current (black) applied to the cell membrane due to sinusoidal voltage stimulation (blue). (**c**) FEM stimulation of deformation distribution of nanofibers on NG at the stress amplitude of 0.5 Mpa.

To estimate the expected behavior of the effective voltage and current applied to the cell membrane by NG, an equivalent circuit was used to model the interaction of the piezoelectric nanofiber NG and cells. Figure 6a shows the diagram of NG-cell and corresponding equivalent circuit model [54,55]. Under external mechanical vibration, the NG deformation produced an external voltage excitation that could be modeled as a voltage source (*V_NG_*). The voltage reached the cell membrane through the culture medium, the conductivity of the medium would affect the voltage that stimulated the cell. The cell membrane is composed of a phospholipid bilayer, which can be regarded as an insulator. The extracellular fluid, cell membrane and intracellular fluid constitute a capacitor (*C_m_*) [56]. The initial potential of ion channel is represented as *V_m_* [57]. Figure 6b shows the simulation results of voltage and current transmitted to the cell membrane through the circuit due to sinusoidal voltage stimulation. When an excitation of 1.3 V was input, the voltage and current delivered to the cell was ~0.8 V and 0.4 mA, respectively. According to the basic principles of the circuit, when *V_NG_* is active, the capacitor *C_m_* is charged (corresponding to the current that is stimulating cell growth), and the voltage across the capacitor (the voltage applied to the cell) *V_c_* (t) (t is time, hereinafter abbreviated as *V_c_*), which is expressed as

$$V_{c}=-\frac{R_{m}V_{NG}}{R_{cm}+R_{m}}\cdot e^{-\frac{R_{cm}+R_{m}}{R_{cm}R_{m}C_{m}}t}+\frac{R_{cm}V_{m}+R_{m}V_{NG}}{R_{cm}+R_{m}}$$

The result calculated by the formula of the voltage applied to the cell was consistent with that of the circuit simulation. 

Since a large deformation of the substrate will hinders cell adhesion, the deformation of aligned nanofibers based on NG under 0.5 MPa stress was analyzed by using finite element model (Figure 6c). The resulting maximum deformation was ~7 µm.

### 3.5. Cell Morphology on NG without Piezoelectric Stimulaton

**Figure 7:**
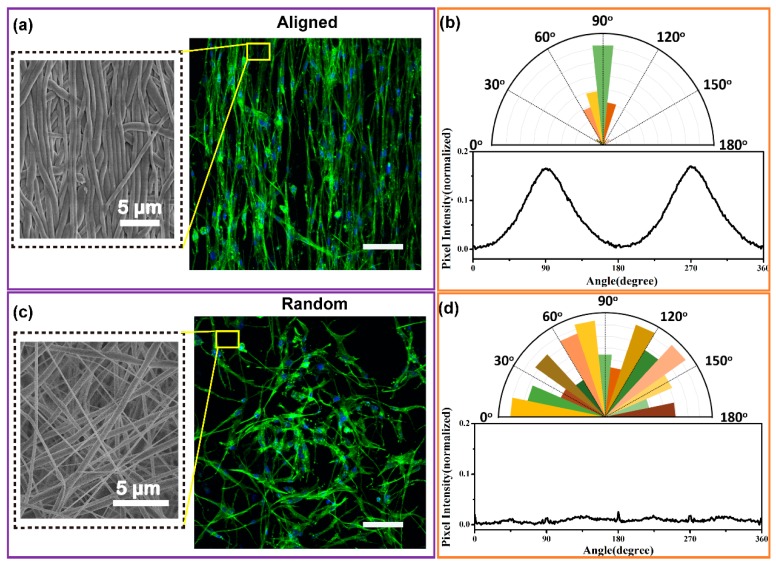
Attachment and alignment of MC3T3-E1 cells on well-aligned and random P(VDF-TrFE) nanofibers after 3 days of culture. (**a**) The SEM image of well-aligned nanofiber substrate (left column) and the confocal fluorescence micrographs of MC3T3-E1 cells (right column). (**b**) 2-D FFT image analysis of cell nuclei (above) and cytoskeleton alignment (below) on well-aligned P(VDF-TrFE) nanofibers. (**c**) SEM image of random nanofiber substrate (left column) and the confocal fluorescence micrographs of MC3T3-E1 cells (right column). (**d**) 2-D FFT image analysis of nuclei (above) and cytoskeleton alignment (below) on random P(VDF-TrFE) nanofibers. F-actin was stained by Alexa Fluor 488-labeled phalloidin (green); cell nuclei were stained by DAPI (blue). The scale bar for confocal fluorescence micrographs is 100 μm.

For tissue engineering, the scaffold material should have excellent cytocompatibility to support cell growth and proliferation [58]. The cell viability data were validated by live/dead kit (Figure S3). The viability was similar between NG and control, suggesting that the P(VDF-TrFE) piezoelectric nanofiber NG has good cytocompatibility. Furthermore, the highly aligned micro-/nanostructure of the fiber-based scaffolds can provide morphological cues for cell attachment and behavioral modulation [59,60,61]. To study the effect of nanofiber morphology on the alignment of cells, the high aligned and random P(VDF-TrFE) nanofibers were used for cell culture. Figure 7a,c shows that the MC3T3-E1 cells attached nicely on both the aligned and random P(VDF-TrFE) nanofibers. The cell cytoskeleton and nucleus showed an elongated morphology on the direction of nanofibers alignment of P(VDF-TrFE) NG, while the MC3T3-E1 cells seeded on random nanofibers displayed a random orientation. This was further verified in that the aligned P(VDF-TrFE) NGs not only have an excellent cytocompatibility but can also guide cell elongation and orientation. 

The results of representative 2-D FFT image are shown in Figure 7b,d. There were two significant symmetrical peaks at 90° and 270° in the plot of aligned P(VDF-TrFE) NGs illustrating that the direction of actin filaments is the same as the nanofibers (Figure 7b below) [62]. The frequency distributions of nuclei were preferentially concentrated along 90° (Figure 7b above) suggesting a specific nuclei orientation. In comparison, the 2-D FFT plot of random samples showed no obvious peaks; the actin filaments were randomly arranged (Figure 7d blow). The arrangement angles of nuclei on the random nanofibers were disordered as the angular histogram exhibits. This confirms that the cell cytoskeleton orientation is caused by the well-aligned surface topography of P(VDF-TrFE) NGs. The results demonstrate that P(VDF-TrFE) NGs can provide not only electrical stimulation signals but also morphologic cues.

### 3.6. Effect of Piezoelectric Stimulation Induced by NG on MC3T3-E1 Cells 

**Figure 8:**
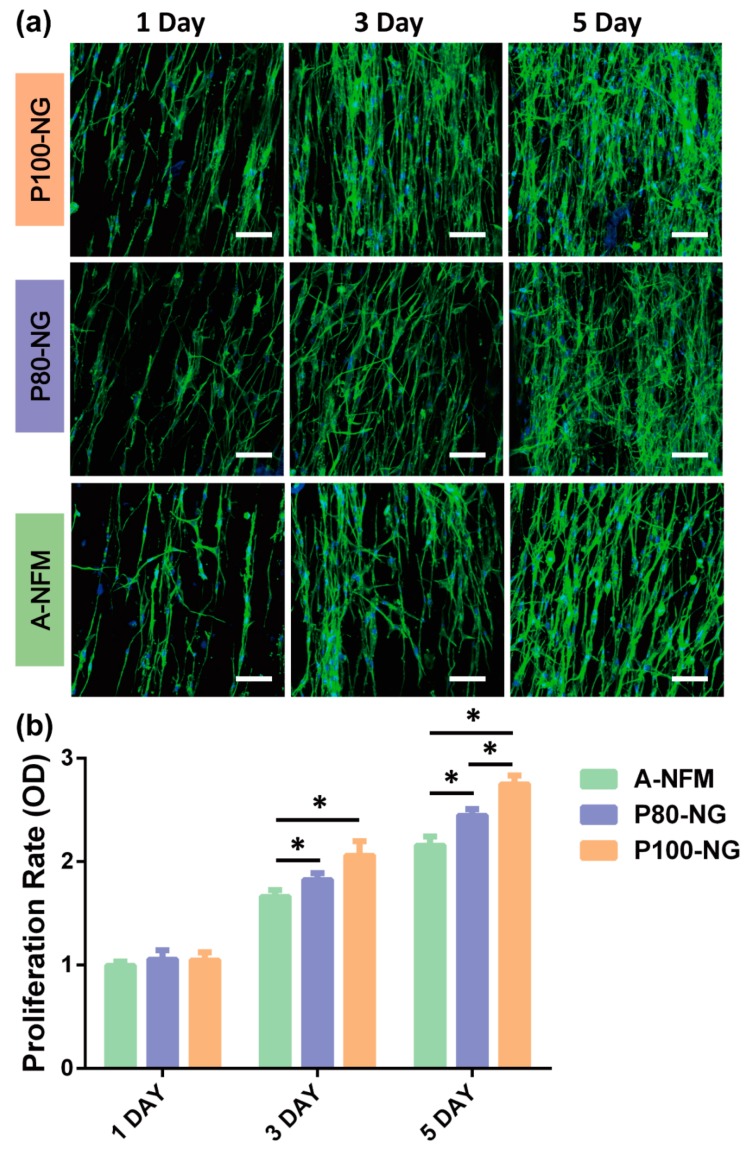
Proliferation of MC3T3 cells on P80-NG, P100-NG, and control A-NFM. (**a**) Fluorescence microscopy images of MC3T3 cells on A-NFM, P80-NG, and P100-NG. (**b**) MC3T3 cells proliferation analyzed by ImageJ software after 1, 3, and 5 days culture. All data represent the mean standard deviation (*n* = 3, * *p* < 0.05). The scale bar is 100 μm.

We next verified the effect of the piezoelectric response of the P(VDF-TrFE) NG acted as electrical stimulus on the cell behavior under dynamic mechanical vibration. To further illustrate the effect of the accurate electrical stimulation modulated by NG on cell proliferation. Here, P100-NG and P80-NG were the experimental groups, and A-NFM was the control. The vibration frequency was set at 2 Hz for mimicking low-frequency biomechanics. The cell proliferation was quantitatively assessed by the CCK-8 assay to estimate the metabolic activity of the total number of MC3T3-E1 cells. Figure 8a shows that the MC3T3-E1 had an elongated morphology along the direction of the nanofibers for all the samples. There was no obvious difference in morphology between cells grown on the two membranes. 

The cell counts of all groups increased on days 1, 3, and 5. The CCK-8 assay showed that the cells grown on both the P100-NG and P80-NG had the higher proliferation rate than those grown on A-NFM illustrating that the piezoelectricity increased cell proliferation (Figure 8b). On day 3, the MC3T3-E1 cells proliferation on the P100-NG and P80-NG was enhanced by 1.24- and 1.10-fold versus that of A-NFM, respectively. No significant difference was observed between the P100-NG and P80-NG. There were significant statistical differences in proliferation rate between cells on P100-NG, P80-NG and A-NFM on day 3 and 5. On day 5, the cells proliferation on the P100-NG and P80-NG was 1.27- and 1.13-fold versus that of A-NFM, respectively; there were significant statistical differences in proliferation rate between cells on P100-NG and P80-NG. A preliminary in vitro assay suggested that piezoelectric stimulations induced by the P(VDF-TrFE) NGs are suitable for the biopotential of MC3T3-E1 cells and can significantly promote cell growth. These results indicate that the combination of customizable exact piezoelectric stimulation and aligned nanostructured NG show the potential application to meet the demand of electrical stimulation according to specific tissue repair. 

## 4. Conclusions

In conclusion, we introduced a promising strategy of a self-powered well-aligned P(VDF-TrFE) piezoelectric nanofiber nanogenerator as an exact piezoelectric stimulator for bone tissue engineering. We investigated the specific effects of post-treatments on the properties of NG. Poling post-treatment could effectively improve the mechanical, piezoelectric, and sensing performances of NG. We also measured the accurate piezoelectric response of NG and emulated the real scene of the electrical stimulation of the cells in vitro during the dynamic status. The well-aligned piezoelectric P(VDF-TrFE) NGs with different encouraged the MC3T3-E1 cells to proliferate in vitro under a sustainable piezoelectric stimulus. To clarify the effect of the exact electrical stimulation on the proliferation fate of preosteoblasts, two different output voltages of NG as stimulators were compared. Our work provides additional insights into the application of P(VDF-TrFE) piezoelectric nanofiber NG as self-powered electrical stimulation system for assisting tissue repair and regeneration.

## Figures and Tables

**Table 1:** Electric properties of P(VDF-TrFE) NFMs with different postprocessing steps at an electric field of 160 MV/m.

Sample	Ec (MV/m)	Ps (mC/m^2^)	Pr (mC/m^2^)	d_33_ (pC/N)	d_31_ (pC/N)	R^2^
U-NFM	60.9	37.9	17.1	0	0.03	0.3496
A-NFM	65.2	43.1	26.9	0	0.07	0.9948
P-NFM	88.1	44.1	32.6	−31	22.88	0.9997

## Supplementary Materials

The following are available online at https://www.mdpi.com/2079-4991/9/3/349/s1, Figure S1: The diameter distribution and morphology, Figure S2: The strain-stress plots of the different NFMs, Figure S3: The cell viability of MC3T3-E1 on P80-NG, P100-NG and control.
