# Supplementary material for: Self-Powered Well-Aligned P(VDF-TrFE) Piezoelectric Nanofiber Nanogenerator for Modulating an Exact Electrical Stimulation and Enhancing the Proliferation of Preosteoblasts
Source: Nanomaterials (Basel). 2019 Mar 3;9(3):349. doi: 10.3390/nano9030349 (PMC6473961; doi:10.3390/nano9030349)
Supplement: Supplementary file 1 [file nanomaterials-09-00349-s001.pdf]

Supporting Information

Self-powered Well-aligned P(VDF-TrFE) Piezoelectric Nanofiber Nanogenerator for Modulating an Exact Electrical Stimulation and Enhancing the Proliferation of Pre-osteoblasts

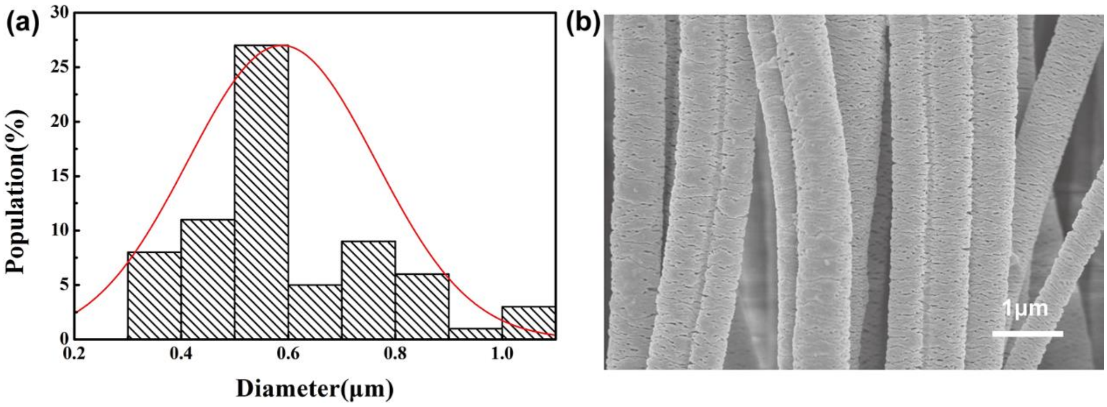

Figure S1. The diameter distribution and morphology. (a) The diameter distribution of electrospun nanofibers; (b) The SEM magnification of A-NFM.

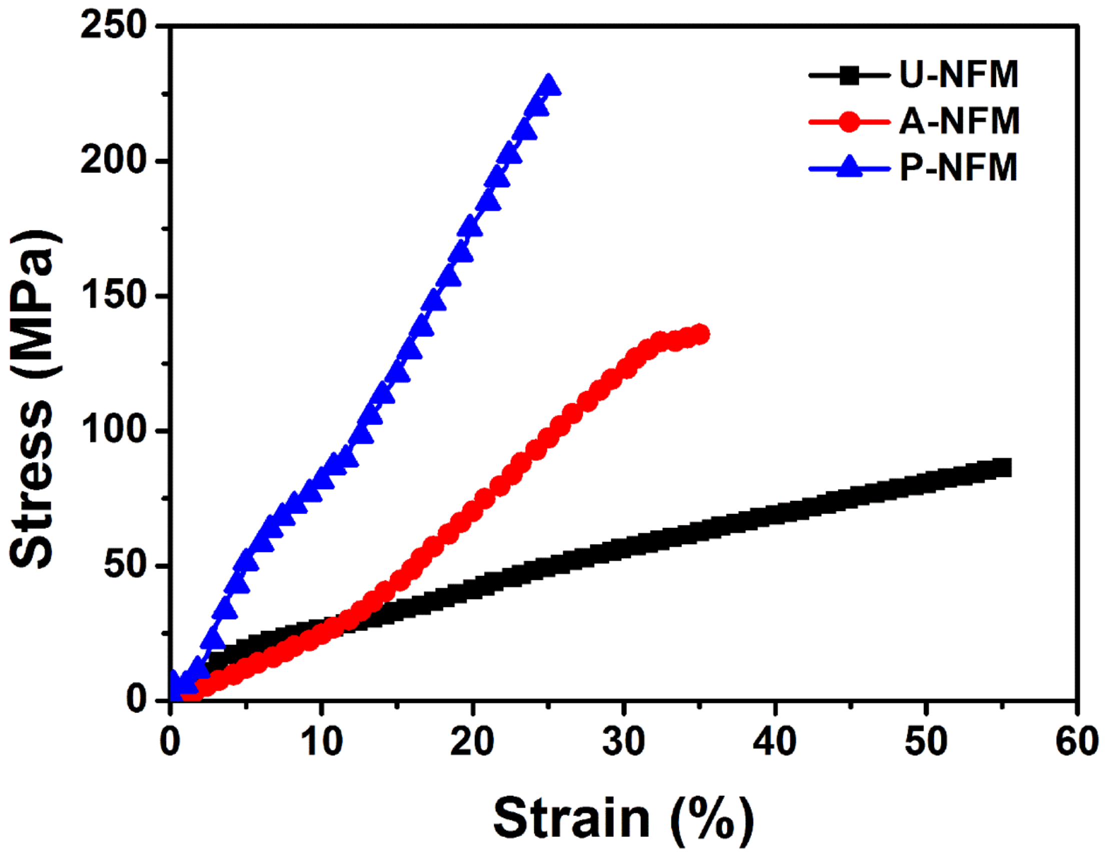

Figure S2. The strain-stress plots of the different NFMs.

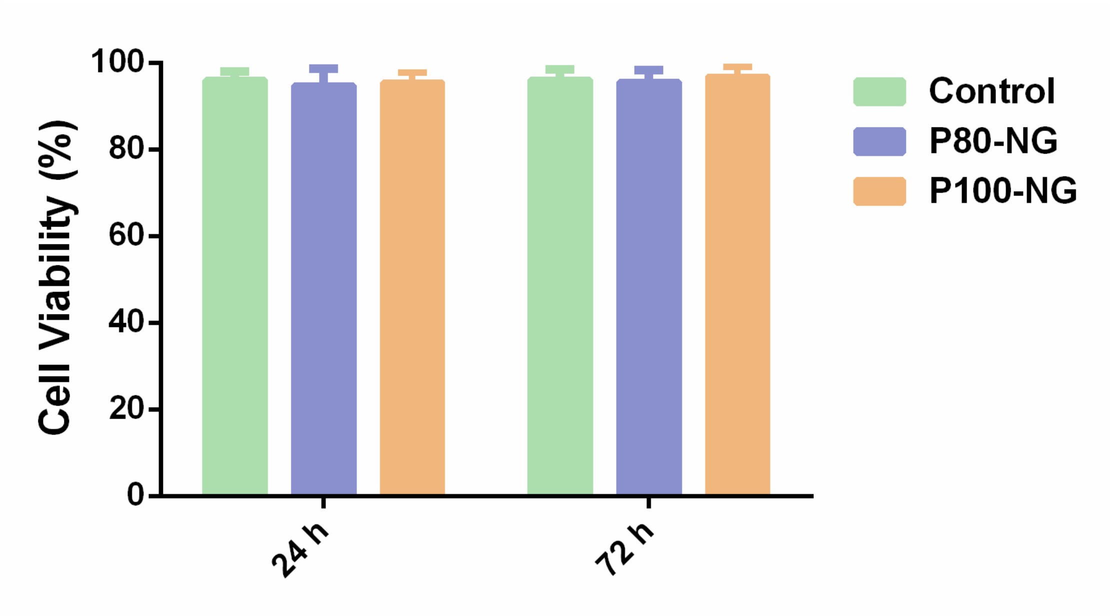

Figure S3. The cell viability of MC3T3-E1 on P80-NG, P100-NG and control (the tissue culture polystyrenes, TCPS).
